# A cost-effective dual reporter system in *Nicotiana benthamiana*

**DOI:** 10.3389/fpls.2026.1732683

**Published:** 2026-02-03

**Authors:** Zeru Zhang, Shuangle Yan, Fuqi Li, Zhijuan Rao, Fahong Fan, Haibin Ren, Yanlin Qu, Ke Yang, Chuanyu Ma, Chen Zhuang, Chentao Lin, Gaoping Qu

**Affiliations:** 1 Basic Forestry and Plant Proteomics Research Center, Haixia Institute of Science and Technology, Fujian Agriculture and Forestry University, Fuzhou, China; 2 Syngenta Biotechnology China, Beijing, China

**Keywords:** dual reporter assay, fluorescence protein, GFP, luciferase, nanoluc, protein quantification

## Abstract

Dual reporter assay has been widely used for quantification of gene expression in various model systems. However, the assay is dependent on commercial kits, which may be inhibitory for many low-budget academic laboratories or high throughput screening. Here, we systematically characterized seven commonly used or potential reporters, and identified NanoLuc-GFP pair as the optimal kit-independent and accurate dual reporter in *Nicotiana benthamiana*. The NanoLuc-GFP system exhibited high stability, which can largely reduce the interference caused by biological variability and dramatic environmental fluctuations. Furthermore, it exhibits greater sensitivity compared to the conventional FLuc/RLuc system commercially available.

## Introduction

Dual reporter assay was first reported in 1996 (Sherf et al., 1996), one luciferase from firefly *Photinus pyralis* (de Wet et al., 1987), FLuc (61 kDa), was selected as the primary reporter for protein quantification, and the other luciferase from *Renilla reniformis* (Lorenz et al., 1991), RLuc (36 kDa), was used as internal control to correct the errors resulting from experimental conditions, such as cell numbers, transfection efficiencies, individual differences and so on. The ratio of FLuc/RLuc was used to characterize the activity of transcription factor/regulator, promoter activity, cis- or trans-regulatory elements and so on. However, FLuc detection is subject to three key limitations that render it kit-dependent. First, the reaction requires not only substrate luciferin but also ATP and Mg^2+^. Second, reaction product oxyluciferin and particularly reaction by-product dehydroluciferyl adenylate (L-AMP) strongly inhibit FLuc activity (Airth et al., 1958; Fraga et al., 2005, 2006; Ribeiro and Esteves da Silva, 2008). Third, FLuc exhibits poor stability (Millar et al., 1992; Thompson et al., 1991). As a result, additives such as coenzyme A (Airth et al., 1958; Fraga et al., 2005; Hasegawa et al., 2020; Sherf et al., 1996), and luciferin analog phenylbenzothiazole (Thompson et al., 1991) are commonly included in the reaction, complicating the detection process.

Since 1996, dozens of smaller luciferases have been characterized. For example, GLuc (20 kDa), derived from the marine copepod *Gaussia princeps*, is a naturally secreted luciferase, which facilitates its enzyme activity detection (Verhaegent and Christopoulos, 2002; Wurdinger et al., 2008); NanoLuc (19 kDa), an engineered luciferase from the deep sea shrimp *Oplophorus gracilirostris*, exhibits the highest brightness (Hall et al., 2012); Luxsit-i (14 kDa, hereafter renamed as iLuc) is a *de novo* luciferase developed using deep learning (Yeh et al., 2023). Some have been used in dual reporter assay (Heise et al., 2013; Wider and Picard, 2017). Compared with these chemiluminescent proteins, fluorescent proteins emit light without requiring additional substrates, making them convenient reporters for protein quantification. Several fluorescent proteins have been reported for protein quantification, such as GFP (Hack et al., 2000), mCherry (Wider and Picard, 2017). Although additional dual reporter systems have been reported (Heise et al., 2013; Wider and Picard, 2017; Wu et al., 2007), the FLuc/RLuc system remains the most widely used. A key reason is the absence of simultaneous, systematic comparisons.

To develop an accurate and cost-effective dual reporter system in *Nicotiana benthamiana*, we systematically investigated the characteristics of four luciferases, RLuc, GLuc, iLuc, NanoLuc, and three fluorescent proteins, GFP, ZsGreen and TagRFP-T. Among the detected seven reporters, only RLuc, NanoLuc and GFP exhibit linearity within the current detected ranges. However, the signal half-lives of RLuc at high concentrations are less than 10 minutes, while NanoLuc has signal half-lives over ten times longer, indicating that NanoLuc is the optimal reporter. Linear reporter GFP, which needs no additional substrate, is the easiest to be detected. Consequently, we preferred NanoLuc and GFP as the optimal reporters. The cost of the novel dual reporter assay is only ~6% of that of the conventional kit-dependent FLuc/RLuc system. Furthermore, the cost-effective dual reporter assay is very stable and can largely reduce the interference caused by the biological and dramatic environmental variations. Moreover, it exhibits greater sensitivity compared to the conventional FLuc/RLuc system commercially available.

## Materials and methods

### Plant materials and growth conditions

*Nicotiana benthamiana* seeds were spread on the surface of moist moss peat. After about 10 days, the germinated seedlings were transplanted to new moist moss peat. Plants were grown at 25°C under long-day conditions with 16 hours of light and 8 hours of darkness. After about three weeks, matured leaves of well-grown plants were selected for agrobacterium infiltration.

### Plasmid construction

To construct a small but high expression vector for protein expressions in *Nicotiana benthamiana*, backbone of pCambia1300 was selected as the base of new vectors. The serial vectors were named as pHEQs, high expression vectors by Qu. After a series of modifications, pHEQ22 was constructed for protein expressions in this paper. The sequence of pHEQ22 was listed in **Supplementary Figure 1**. pHEQ22 contains a 35S promoter followed by a multiple cloning site and three terminators, 35S terminator-NOS terminator-35S terminator. In addition, the T-DNA of both the left border and the right border contain sequences of the long intergenic region (LIR) from bean yellow dwarf virus (BeYDV), as well as a sequence of the short intergenic region (SIR) from BeYDV on the left border.

For generation of pHEQ22-RLuc, pHEQ22 was digested with *Bam*HI/*Stu*I. The *RLUC* was amplified with BamHI-Rluc-F/Rluc-StuI-R from a synthesized DNA template, the PCR product was inserted into the linearized pHEQ22 through seamless-cloning.

For generation of pHEQ22-GLuc, pHEQ22 was digested with *Bam*HI/*Stu*I. The *GLUC* was amplified with BamHI-Gluc-F/Gluc-StuI-R from a synthesized DNA template, the PCR product was inserted into the linearized pHEQ22 through seamless-cloning.

For generation of pHEQ22-NanoLuc, pHEQ22 was digested with *Bam*HI/*Stu*I. The *NANOLUC* was amplified with BamHI-nanoLuc-F/nanoLuc-StuI-R from a synthesized DNA template, the PCR product was inserted into the linearized pHEQ22 through seamless-cloning.

For generation of pHEQ22-iLuc, pHEQ22 was digested with *Bam*HI/*Stu*I. The *ILUC* was amplified with BamHI-iLuc-F/iLuc-StuI-R from a synthesized DNA template, the PCR product was inserted into the linearized pHEQ22 through seamless-cloning.

For generation of pHEQ22-GFP, pHEQ22 was digested with *Bam*HI/*Stu*I. The e*GFP* was amplified with BamHI-GFP-F/GFP-StuI-R, the PCR product was inserted into the linearized pHEQ22 through seamless-cloning.

For generation of pHEQ22-ZsGreen, pHEQ22 was digested with *Bam*HI/*Stu*I. The *ZsGREEN* was amplified with BamHI-ZsGreen-F/ZsGreen-StuI-R from a synthesized DNA template, the PCR product was inserted into the linearized pHEQ22 through seamless-cloning.

For generation of pHEQ22-TagRFP-T, pHEQ22 was digested with *Bam*HI/*Stu*I. The *TagRFP-T* was amplified with BamHI-TagRFP-F/TagRFP-StuI-R from a synthesized DNA template, the PCR product was inserted into the linearized pHEQ22 through seamless-cloning.

For generation of pHEQ22-3×UAS-mini35S-GFP, plasmid pHEQ22 was linearized by PCR into two fragments. Fragment 1 (4192 bp) was amplified with primers f1-1F for UAS/f1-1R for UAS; fragment 2 (5702 bp) was amplified with primers f2-2F for UAS/f2-2R for UAS. The 3×UAS sequence was incorporated into the plasmid by primers f1-1F of UAS and f2-2R of UAS. The two fragments were ligated through seamless-cloning.

For generation of pHEQ22-3×UAS- mini35S-FLuc, pHEQ22-FLuc was constructed first. pHEQ22 was digested with *Bam*HI/*Stu*I. The *FLUC* was amplified with BamHI-FLuc-F/FLuc-StuI-R from a synthesized DNA template, the PCR product was inserted into the linearized pHEQ22 through seamless-cloning. Next, pHEQ22-FLuc was linearized by PCR into two fragments. Fragment 1 (5125 bp)was amplified using primers f1-1F for UAS/f1-1R for UAS; fragment 2 (5702 bp) was amplified with primers f2-2F for UAS/f2-2R for UAS. The two fragments were then ligated through seamless-cloning to generate pHEQ22-pUAS-FLuc.

For generation of pHEQ22-GAL4 BD, pHEQ22 was digested with *Bam*HI/*Stu*I. *GAL4 BD* was amplified with GAL4 BD-F for pHEQ22-BD/VP16/SRDX//GAL4 BD-R for pHEQ22-BD from plasmid pGBKT7, the PCR product was inserted into the linearized pHEQ22 through seamless-cloning.

For generation of pHEQ22-GAL4 BD-VP16, pHEQ22 was digested with *Bam*HI/*Stu*I. Fragment 1 was amplified with GAL4-BD-F for pHEQ22-BD/VP16/SRDX//GAL4-BD-R for pHEQ22-BD-VP16, and fragment 2 was amplified with VP16-F for pHEQ22-BD-VP16/VP16-R for pHEQ22-BD-VP16 from DNA sequence containing VP16. Two fragments were inserted into the linearized pHEQ22 through seamless-cloning.

For generation of pHEQ22-GAL4 BD-SRDX, pHEQ22 was digested with *Bam*HI/*Stu*I. *GAL4 BD-SDRX* was amplified with GAL4 BD-F for pHEQ22-BD/VP16/SRDX//GAL4 BD-R for pHEQ22-BD-SRDX from plasmid pGBKT7, the PCR product was inserted into the linearized pHEQ22 through seamless-cloning.

### Transient expression assay in tobacco leaves

The expression plasmids were individually transformed into *agrobacterium tumefaciens* strain C58C1 through electroporation transformation. The agrobacterium were grown on LB medium plates at 28°C for two days. Single clone was picked and inoculated into 5–15 ml LB liquid medium. The culture was then incubated at 28°C with a shaking speed of 180 rpm. After about 24 hours, agrobacterium was collected by centrifugation. and resuspended by infiltration solution (10 mM MgCl_2_, 10 mM MES (pH 7.4)). The agrobacterium was diluted to an OD_600_ of 0.2 in infiltration solution. Then, 20 mM acetosyringone was added to achieve a final concentration of 200 μM. Kept at room temperature in darkness for at least 3 hours, agrobacterium was infiltrated into tobacco leaves using a 1 ml syringe. For consistent lysis efficiency, samples of almost the same weight (30 ± 2 mg) were collected at the third day after agroinfiltration.

### Protein extraction assay

The protein extraction assay was performed as described previously (Qu et al., 2020). In brief, total proteins were extracted from the collected samples with 800 μl extraction buffer containing 50 mM Tris–HCl (pH 7.5), 150 mM NaCl, 1 mM EDTA, 1% Triton X-100 (v/v), 2% glycerol (v/v) and 1× protease inhibitor cocktail (Roche, #04693159001).

### Luciferase RLU detection assay

RLUs of all detected luciferases were measured in black 96-well flat plate (*n* = 3) using the Spark microplate reader (Tecan). In brief, three types of substrates, coelenterazine (CTZ), diphenylterazine (DTZ), and furimazine were purchased from Coredawn Biotech., and were dissolved in ethanol, DMF and DMSO to prepare 2 mM, 2 mM and 5 mM stock solutions, respectively. Appropriate volumes of substrates were added to 360 μl protein extract containing the corresponding luciferases. The mixtures were then vortexed thoroughly to ensure homogeneous mixing. Subsequently, 100 μl sample was pipetted to a 96-well plate, with each sample having three replicates (*n* = 3). Detections of relative luciferase units were initiated at 180 seconds after the additions of the substrate. The parameters set for RLU were as follows: photos were captured within the wavelength range of 360–700 nm, integration time was set to 1000 ms, and the values are captured photos counts per second.

### Fluorescent protein RFU detection assay

Fluorescent proteins are quantified by relative fluorescent unit (RFU). RFUs of all detected fluorescent proteins were also measured in black 96-well flat plate (*n* = 3) using Spark microplate reader (Tecan).

For GFP, the parameters were as follows: the excitation wavelength was 485 ± 20 nm, the emission wavelength was 535 ± 20 nm, gain manual was 54, number of flashes was 30, and integration time was 40 μs.

For ZsGreen, the parameters were as follows: the excitation wavelength was 490 ± 10 nm, the emission wavelength was 521 ± 20 nm, gain manual was 58, number of flashes was 30, and integration time was 40 μs.

For TagRFP-T, the parameters were as follows: the excitation wavelength was 532 ± 20 nm, the emission wavelength was 588 ± 20 nm, gain manual was 68, number of flashes was 30, and integration time was 40 μs.

### The detection of FLuc/RLuc dual reporter assay

The detection of FLuc/RLuc was performed following the promega E1910 kit manual with minor modifications. Briefly, 30 mg of ground plant power was lysed in 700 μl 1×Passive Lysis Buffer, followed by sonication, 30 seconds total, with 2-second on/3-second off cycles, and incubation on ice for 30 min. After centrifugation, the supernatant was diluted 100 fold with 1×Passive Lysis Buffer. A 20 μl diluted supernatant was added to a black 96-well flat plate, followed by 50 μl LARII. The plate was shaken 5 seconds using Spark microplate reader (Tecan), and FLuc activity was measured according to the protocol mentioned above. Subsequently, 50 μl Stop&Glo reagent was added, the mixture was shaken to combine, and RLuc activity was measured.

### Half-life calculation

Half-life (t_1/2_) values were calculated using the formula, *F_t_* = *F_0_*× e{sp}-{it}kt{/sp}{/it}. The *K* values were determined by taking the natural logarithm on both sides of the equation and generating a scatter plot of ln(RLU) versus time. Subsequently, the half-life values were calculated using the equation, *t*_1/2_ = ln(2)/*K*.

## Results

### Variable effects of substrate concentration on luciferase activity

Luciferase activity is commonly quantified by relative luminescence unit (RLU), which is primarily influenced by two key factors, substrate concentration and enzyme concentration. To investigate these effects, four representative luciferases, RLuc, GLuc, iLuc, and NanoLuc, were selected, and their RLU time-course curves were tested.

**Figure 1:**
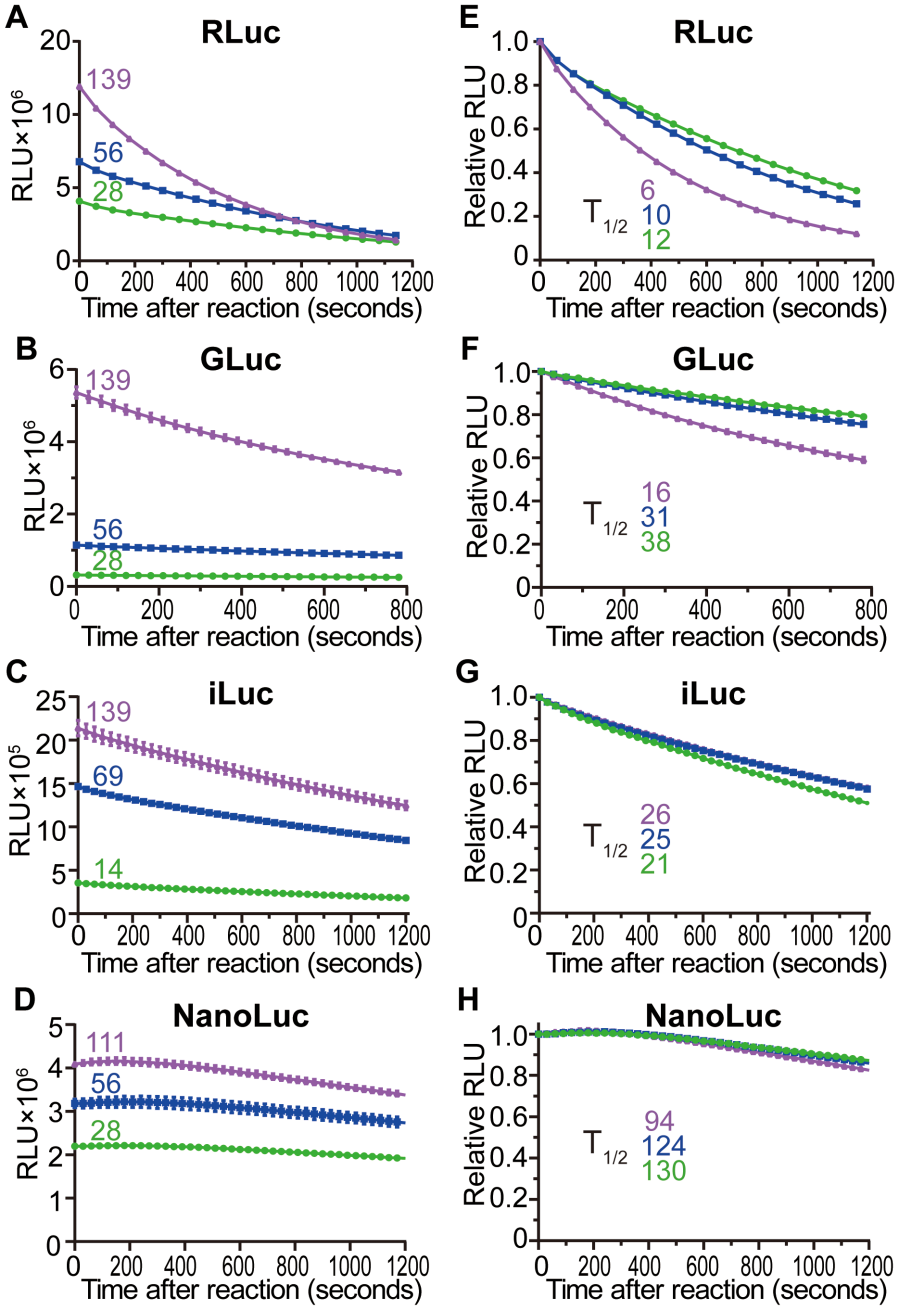
Time kinetics and signal half-lives of luciferases under different substrate concentrations. **(A)** RLuc activities at different coelenterazine (CTZ) concentrations (μM) after reaction. The colored numbers, which correspond to the time course curves, represent different substrate concentrations (the same below). **(B)** GLuc activities at different CTZ concentrations. **(C)** iLuc activities at different diphenylterazine (DTZ) concentrations. **(D)** NanoLuc activities at different furimazine concentrations. **(E–H)** Relative RLUs of RLuc **(E)**, GLuc **(F)**, iLuc **(G)** and NanoLuc **(H)** at different substrate concentrations. The half-lives (t_1/2_) were calculated, the color also corresponded to different substrate concentrations. Values are means ± SD (*n* = 3).

Theoretically, enzyme activity rises with increasing substrate concentration until saturation. Consistent with the expectation, our results showed that RLUs of the four tested luciferases increased with rising substrate concentration (**Figures 1A–D**). However, no saturation phenomenon was observed, which might be attributed to insufficient substrate concentration.

On the other hand, enzymatic activity should be stable with excessive substrate. However, the RLUs of both RLuc and GLuc appeared to become more unstable as the substrate concentration increased. To directly compare the stability, RLUs were normalized to their respective values at 0 seconds, and signal half-lives were calculated. Surprisingly, RLuc, the most commonly used reporter, exhibited the shortest half-life among the four luciferases tested (**Figures 1E–H**). Furthermore, its half-life decreased as the concentration of substrate coelenterazine (CTZ) increased (**Figure 1E**), which may result from inhibition by the reaction product, coelenteramide (Hart et al., 1979; Verhaegent and Christopoulos, 2002). Specifically, the half-lives of RLuc were 12 min, 10 min and 6 min in the presence of 28 μM, 56 μM and 139 μM CTZ, respectively. GLuc also uses CTZ as a substrate, and likely due to a similar mechanism, its half-life similarly decreased with increasing substrate concentration (**Figure 1F**). The signal half-lives of GLuc were 38 min, 31 min and 16 min in the presence of 28 μM, 56 μM and 139 μM CTZ, respectively. In contrast, iLuc uses diphenylterazine (DTZ) as its substrate, while NanoLuc uses furimazine. Importantly, RLUs of both iLuc and NanoLuc exhibit almost uniform stability across the tested substrate concentrations (**Figures 1G, H**). For iLuc, half lives were 21 min, 25 min and 26 min at 14 μM, 69 μM and 139 μM DTZ, respectively. NanoLuc exhibits the longest half-lives, with values of 130 min, 124 min and 94 min recorded at 28 μM, 56 μM and 111 μM furimazine, respectively. These results suggest that for RLuc and GLuc, substrate concentration should not be excessively high, whereas for iLuc and NanoLuc, substrate concentration has no significant impact on the results.

Collectively, substrate concentration exerts variable effects across different luciferases, and an optimal substrate concentration is critical for reliable RLU detection.

### The luminescence signal half-life is negatively correlated with luciferase concentration

Based on the magnitude and stability of the luminescence signal (**Figure 1**), the optimal substrate concentrations were set at 28 μM for RLuc, GLuc and NanoLuc, and 97 μM for iLuc. To investigate how signal half-life is influenced by luciferase concentration, we tested the time-course curves for a serial dilution-based luciferase concentration gradient, with the highest concentration defined as 100%.

**Figure 2:**
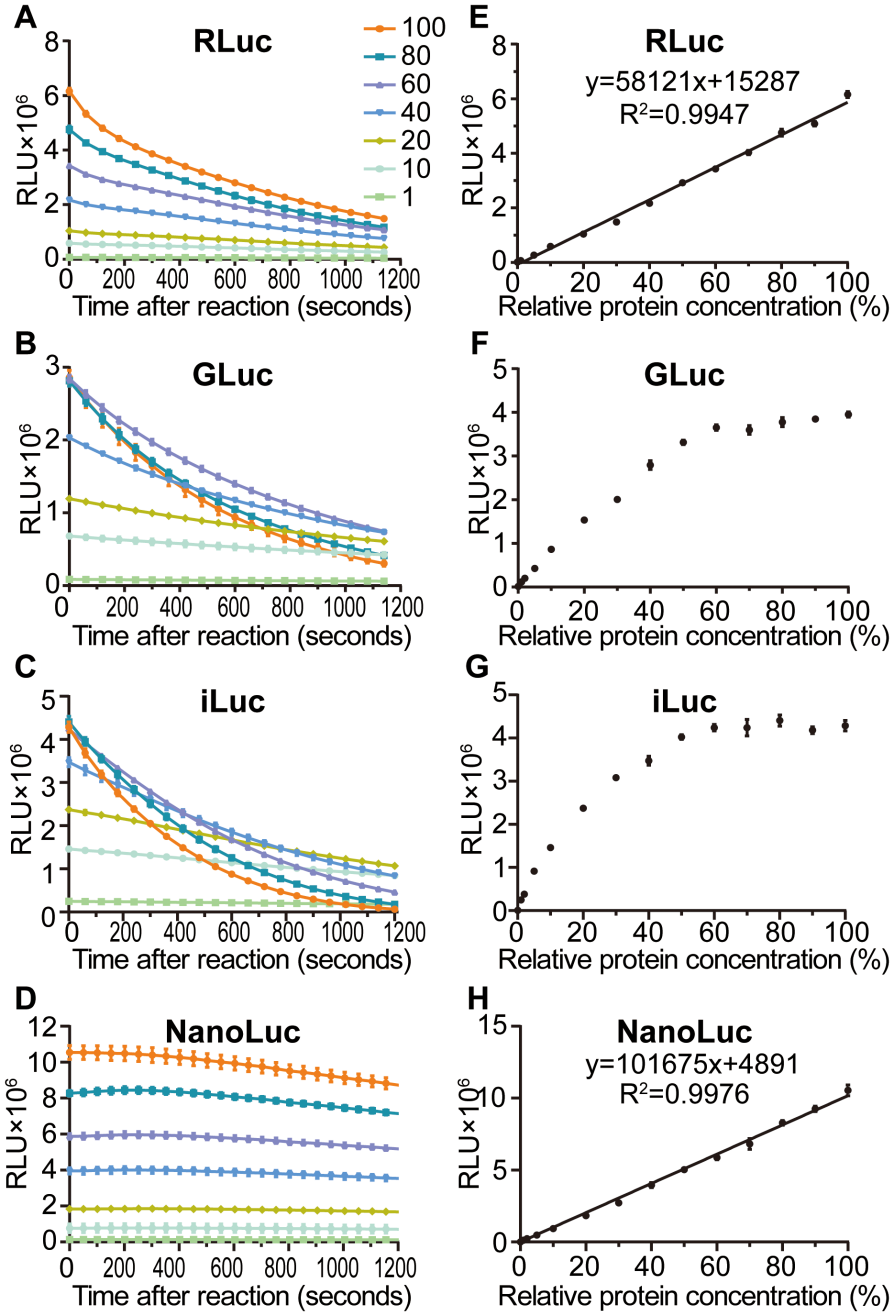
Time kinetics of luciferases at different enzyme concentrations. **(A)** RLuc activities at different enzyme concentrations through serial dilution after reaction (the same below). CTZ concentration was 28 μM. **(B)** GLuc activities at different enzyme concentrations. CTZ concentration was 28 μM. **(C)** iLuc activities at different enzyme concentrations. DTZ concentration was 97 μM. **(D)** NanoLuc activities at different enzyme concentrations. Furimazine concentration was 28 μM. **(E-H)** Standard curve of RLuc **(E)**, GLuc **(F)**, iLuc **(G)** and NanoLuc **(H)**. Data correspond to those presented in A-D at 0 seconds. The numbers indicate relative luciferase concentrations (%). Values are means ± SD (*n* = 3).

RLUs of both RLuc and NanoLuc increased proportionally with higher enzyme concentrations (**Figures 2A, D**). In contrast, for GLuc (**Figure 2B**) and iLuc (**Figure 2C**), the increased RLUs were only observed at lower enzyme concentrations, below 60%, with nearly identical RLUs across the 60%-100% concentration range. And as the reaction proceeded, RLUs declined more markedly at higher enzyme concentrations, resulting in lower RLUs for samples with higher luciferase abundance (**Figures 2B, C**).

To directly compare the effect of luciferase concentration on RLUs, signal half-lives were calculated. The signal half-lives of all tested luciferases decreased with increasing enzyme concentration (**Figure 2** and **Table 1**), partly due to insufficient substrate availability at high luciferase concentrations. Notably, RLuc exhibited the shortest signal half-life at concentrations below 20%. In contrast, and consistent with a previous report (Hall et al., 2012), NanoLuc activities remained highly stable across all detected enzyme concentrations and displayed the longest signal half-life, 86–304 min, which is almost 10 fold longer than that of the other three luciferases tested. From the time-course curves, it can be found that RLUs of both GLuc and iLuc decreased faster at high concentrations (**Figures 2B, C**). The signal half-lives of GLuc and iLuc at 100% relative concentration were 6.1 min and 3.8 min, respectively, corresponding to only 15.8% and 8.3% of their signal half-lives at 1% relative concentration. Such significant shortening of signal half-lives at high enzyme concentrations could lead to erroneous protein quantification.

**Table 1:** The signal half-lives of luciferases.

Relative protein concentration (%)	Half-life (t_1/2_, min)			
	RLuc	GLuc	iLuc	NanoLuc
100.0	9.0	6.1	3.8	86.9
80.0	9.5	7.0	4.8	132.8
60.0	11.7	9.8	6.8	160.5
40.0	13.1	12.8	10.3	172.4
20.0	16.0	19.4	18.3	231.0
10.0	17.6	27.8	26.5	256.7
1.0	23.3	38.5	45.7	304.0

### The standard curves of reporters

To directly assess the linear range, RLUs measured at 0 seconds were used to establish the standard curves (**Figure 2**). Consistent with previous reports (Hall et al., 2012; Sherf et al., 1996), activities of both RLuc and NanoLuc exhibited linearity across the detected enzyme concentration ranges (**Figures 2E, H**). Additionally, their absolute concentrations at the 100% relative protein concentration were quantified via the Bradford assay (**Supplementary Figure 2**), with RLuc and NanoLuc determined to be 17 nM and 0.275 nM, respectively. And the measured concentrations and corresponding RLUs are comparable to the published results (Hall et al., 2012). However, due to the significant shortening of half-lives at high enzyme concentrations, GLuc activities showed linearity only at low concentrations (**Figure 2F**), which is contrary to the broad linear range reported previously (Verhaegent and Christopoulos, 2002), which might be attributed to variations in CTZ and GLuc concentrations. Similarly, iLuc activities displayed linearity only at low concentrations (**Figure 2G**). Combined with the time-course curve results (**Figures 2B, C**), relative lower luciferase concentrations should be employed for detecting GLuc and iLuc.

**Figure 3:**
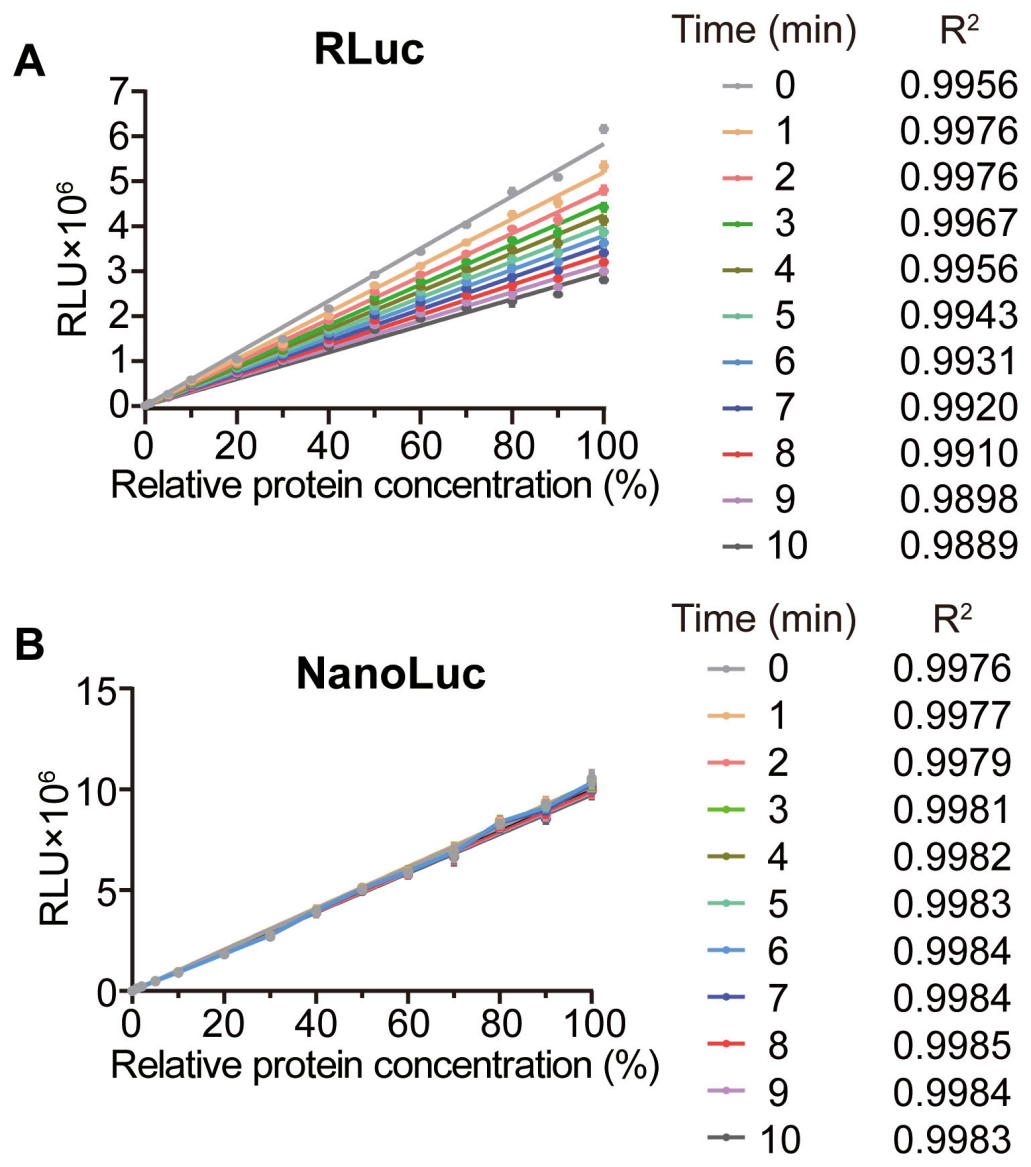
Standard curves of linear reporter RLuc and NanoLuc at different reaction time. **(A, B)** Standard curves of RLuc **(A)** and NanoLuc **(B)** at different reaction time. Data correspond to those presented in **Figure 2A** and **2D** at 0–60 seconds. All values are means ± SD (*n* = 3).

Although the standard curves of both RLuc and NanoLuc were linear, the signal half-life of RLuc was less than one tenth that of NanoLuc (**Table 1**). To assess the possible effect, we redrew their standard curves at different reaction time points. The slopes of RLuc’s standard curves decreased with prolonged reaction time, with the most notable reduction occurring within the first two minutes (**Figure 3A**). So, RLuc detection should be done at the same reaction time point, which undoubtedly increases the complexity of the assay. In contrast, NanoLuc’s standard curve slopes remained nearly constant during the tested ten minutes (**Figure 3B**). These results suggest that NanoLuc performs as an optimal reporter compared to RLuc.

**Figure 4:**
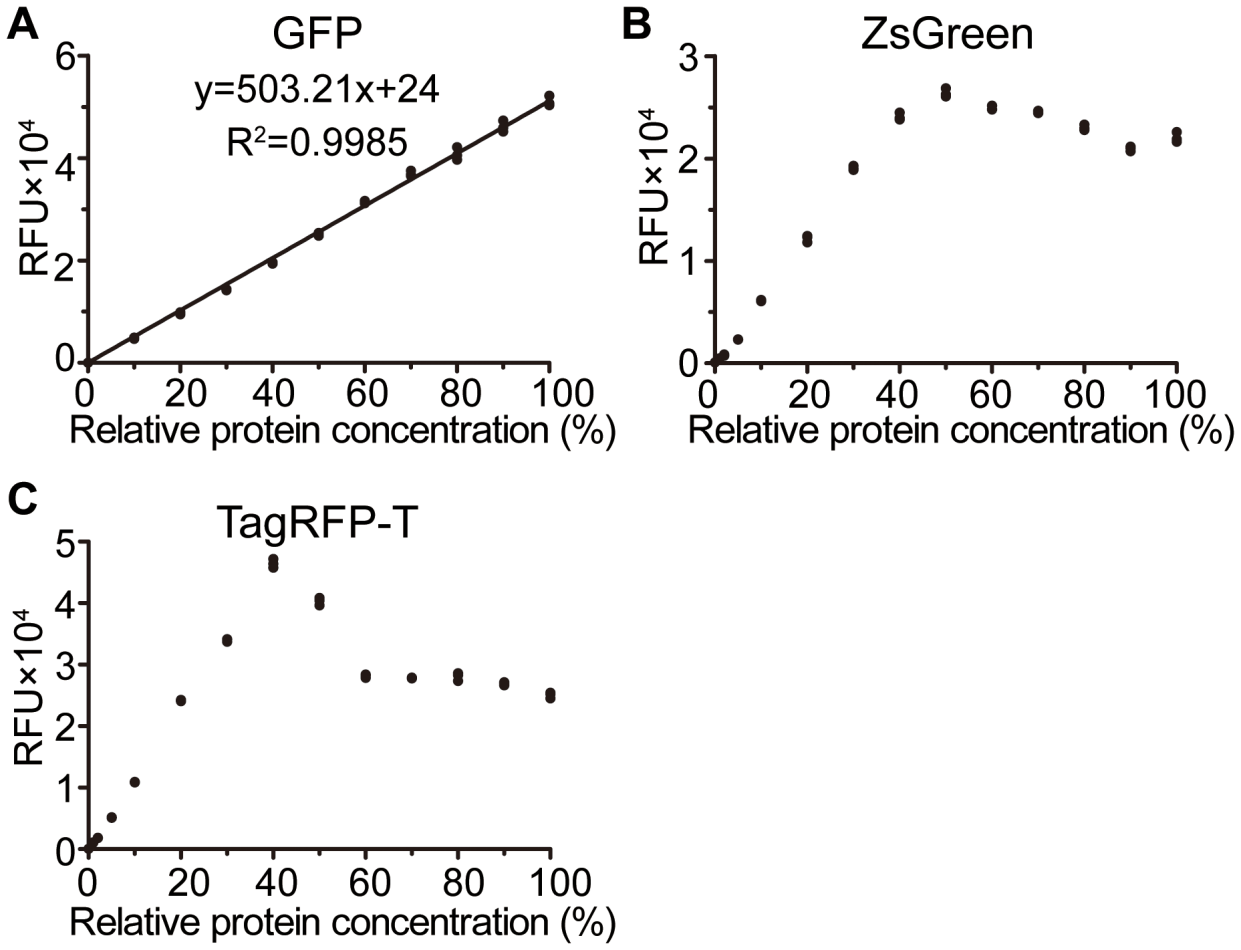
Linearities of fluorescent proteins. **(A–C)** Standard curves of fluorescent protein GFP **(A)**, ZsGreen **(B)**, and TagRFP-T **(C)**. Values are shown as aligned with each replicate (*n* = 3).

In addition to luciferases, we also evaluated the potential of fluorescent proteins as reporters by examining their linearities across serial dilution. Three candidates were selected: the monomeric fluorescent protein GFP (27 kDa) and TagRFP-T (28 kDa), as well as the tetramer fluorescent protein ZsGreen (26 kDa). However, only GFP exhibited linearity across the entire detected concentration range (**Figure 4A**), with the absolute concentration at the 100% relative concentration determined to be 1125 nM (**Supplementary Figures 2C, D**). In contrast, both ZsGreen (**Figure 4B**) and TagRFP-T (**Figure 4C**) showed linearity exclusively at low concentrations. The nonlinearity observed for ZsGreen may be attributed to its oligomerization (Yanushevich et al., 2001), while that of TagRFP-T could result from disrupted equilibrium states of its different isomers at high concentrations (Liu et al., 2016).

For fluorescent proteins, spontaneous fluorescence of plant materials, such as chlorophyll, cannot be neglected. To address this issue, spontaneous fluorescence intensities were measured across a gradient of fresh weights (**Supplementary Figure 3**). The spontaneous fluorescence of the protein extraction buffer alone was approximately 20-50, which is negligible, and the spontaneous fluorescence of plant materials were approximately 200, 500, and 150 for GFP, TagRFP-T and ZsGreen, respectively. Interestingly, their spontaneous fluorescence was almost unaffected by variations in fresh weight. Given that its RFU value was ~50,000 at the 100% relative protein concentration, GFP exhibited a maximum signal-to-background ratio of approximately 2000, comparable to those of the tested luciferases. These results suggest that GFP also performs as an optimal reporter for protein quantification.

### Establishment of a kit-independent dual reporter system in *Nicotiana benthamiana*

Based on the detected parameters, such as linearity, signal intensity and decay rate, we preferred NanoLuc and GFP as optimal reporters. To test the stability of this dual reporter system, we employed GFP as the primary reporter and NanoLuc as an internal control in a widely used tobacco transient protein expression system.

**Figure 5:**
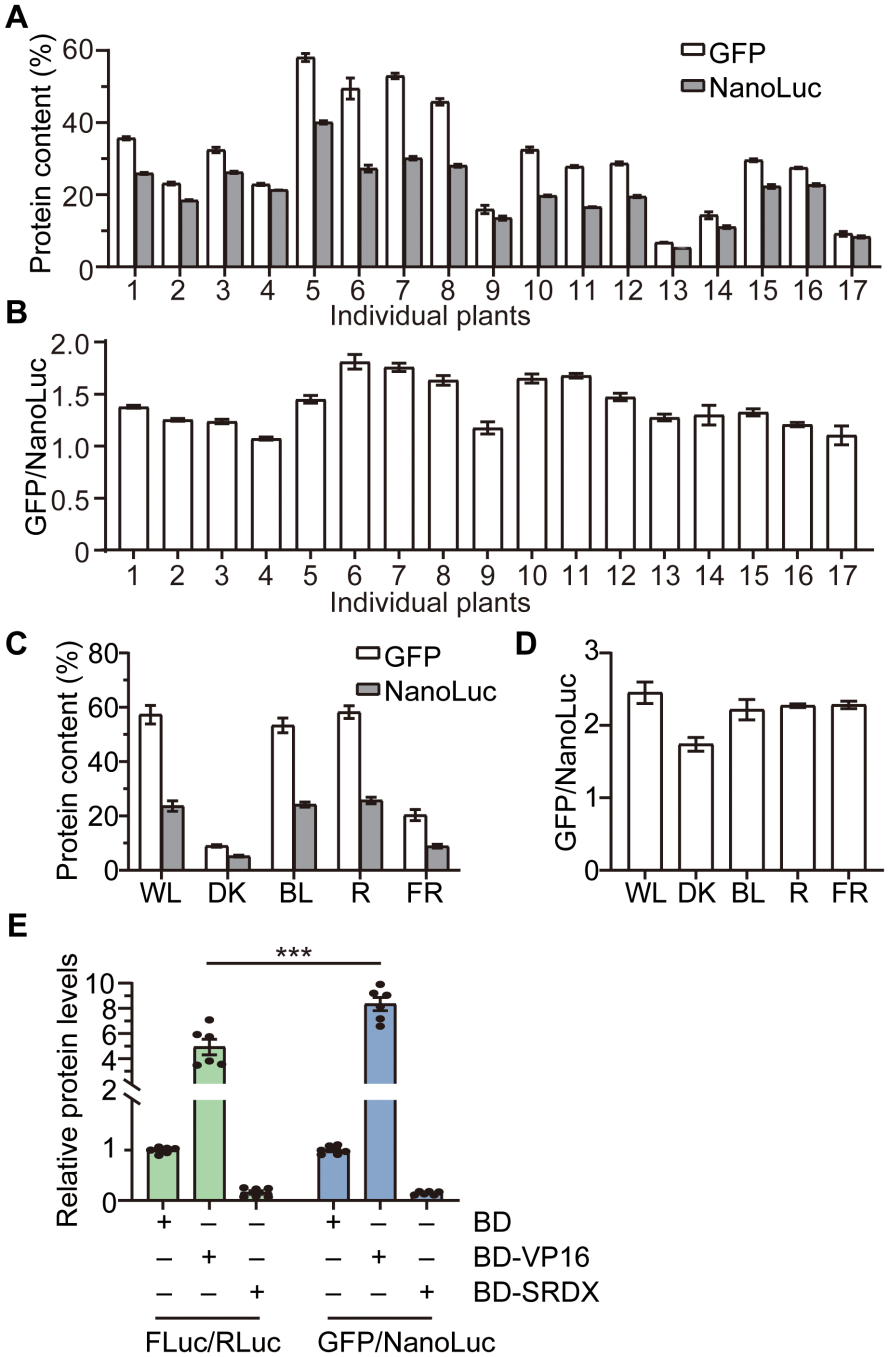
Characterization of the kit-independent dual reporter system. **(A)** Protein expressions of GFP and NanoLuc in different individual plants. Seventeen tobacco plants were randomly selected for transient protein expression. The agrobacterium concentrations for both GFP and NanoLuc were adjusted to 0.2 (OD_600_). Values are means ± SD (*n* = 3). **(B)** The ratio of GFP/NanoLuc (data from A). **(C)** Protein expressions of GFP and NanoLuc in different monochromatic light. The agrobacterium concentrations for GFP and NanoLuc were adjusted to 0.2 and 0.1, respectively. Tobacco plants were illuminated in different light conditions after infiltration. Values are means ± SE (*n* = 4). WL: white light; DK: darkness; BL: blue light; R: red light; FR: Far-red light. **(D)** The ratio of GFP/NanoLuc (data from C). **(E)** Comparison of GFP/NanoLuc with the gold dual reporter system FLuc/RLuc. The NanoLuc RLUs and GFP RFUs were converted to relative protein contents according to the corresponding standard curves in 2H and 4A, respectively. Two-tailed Student’s *t*-test was used to analyze the significant difference. ***P < 0.001.

First, we assessed the system’s stability across different individuals. Seventeen tobacco plants were randomly selected and subjected to agrobacterium infiltration. On the third day post-infiltration, equal amounts of tissue were harvested to ensure uniform lysis efficiency during protein extraction, followed by quantification of reporter gene expression. Using standard curves for GFP (**Figure 4A**) and NanoLuc (**Figure 2H**), signal intensities were converted to relative protein quantities. Results revealed substantial variability in GFP and NanoLuc expression across individual leaves, with some differences exceeding 8.6 fold for GFP and 7.5 fold for NanoLuc (**Figure 5A**). However, GFP/NanoLuc ratios remained nearly consistent, with a maximum deviation of less than 69%, and a standard error of 0.04 (**Figure 5B**), indicating that the dual reporter system can effectively mitigate the impact of biological variability using biological repeats.

Second, we evaluated the system’s stability under varying environmental conditions. Given that light is a critical environmental factor for plants, we tested the stability across different light conditions. Based on the notably high NanoLuc values observed above, we reduced the agrobacterium concentration from 0.2 to 0.1 (OD_600_). Following agrobacterium infiltration, plants were exposed to different monochromatic light conditions. In line with the fact that plants primarily absorb blue and red light for photosynthesis, protein levels in blue light (BL) and red light (R) were comparable to those in white light (WL). In contrast, protein levels in darkness (DK) and far-red light (FR) were approximately one-sixth and one-third of those in visible light, respectively (**Figure 5C**). Nevertheless, GFP/NanoLuc ratios varied by less than 10% across WL, BL, R, and even FR conditions (**Figure 5D**). Notably, continuous darkness exceeding 48 hours might trigger plant stress responses, resulting in more pronounced ratio differences compared to light conditions. However, the maximum disparity, observed between WL and DK, remained less than 30% (**Figure 5D**). These results confirmed that the dual reporter system can largely reduce the interference caused by dramatic environmental variations.

### Comparison of GFP/NanoLuc and FLuc/RLuc systems

Next, we compared the performance of the kit-independent GFP/NanoLuc dual reporter system with that of the widely used kit-dependent FLuc/RLuc system. Plasmids were constructed where the primary reporters GFP and FLuc were each placed under the control of a mini35S promoter with 3×GAL4 UAS (upstream activation sequence) upstream (Morsut et al., 2016), while the internal controls NanoLuc and RLuc were driven by the constitutive 35S promoter. The effector proteins used were BD-fused VP16, a transcription activator, and BD-fused SRDX, a transcription repressor. The experiment included six biological repeats from two distinct time points.

Consistent with the known activation and repression activities of VP16 and SRDX, respectively, both dual reporter systems showed significant alterations in their reporter-to-internal control ratios in response to effectors. Specifically, the FLuc/RLuc ratio was activated 4.9 fold by VP16, while the GFP/NanoLuc ratio exhibited a more robust 8.3 fold activation under the same condition. For repression, the FLuc/RLuc ratio was reduced to 0.16 fold by SRDX, and the GFP/NanoLuc ratio was similarly reduced to 0.15 fold. These results indicate that the kit-independent GFP/NanoLuc dual reporter system exhibits higher sensitivity than the conventional kit-dependent FLuc/RLuc system.

## Discussion

### The GFP-NanoLuc dual reporter assay is accurate and cost-effective

The novel dual reporter assay, GFP-NanoLuc, is kit-independent and very stable, which can largely reduce the interference caused by the variation of individuals and environments (**Figure 5**). For example, compared with that in WL, BL or R, the expression level of the reporter GFP in DK has decreased by approximately fivefold, which far exceeded the range of experimental error, but the ratio of GFP/NanoLuc in DK is almost similar to that in WL, BL or R. In addition, the established dual reporter system is also applicable to other systems, such as *E.coli*, yeast, and mammalian HEK293T cells, even without cell lysis.

The GFP/NanoLuc system exhibited higher sensitivity than the conventional FLuc/RLuc dual reporter system. Compared with the FLuc/RLuc system, the GFP/NanoLuc system not only showed 1.7 fold greater sensitivity to the effector protein VP16, but also showed smaller standard errors (**Figure 5**).

The GFP/NanoLuc system is more cost-effective than the FLuc/RLuc system. Specifically, the cost of GFP/NanoLuc system is less than $0.08 per reaction, whereas the cost of FLuc/RLuc system is $1.23 per reaction, even using only half the dosage recommended in the kit manual. In addition, the unit price of the NanoLuc substrate is 30 times higher than that of the FLuc substrate. In the future, with the widespread application of the GFP/NanoLuc system, the cost is expected to decrease by at least 50%, and even by 90%. As a result, the GFP/NanoLuc would significantly reduce the cost of dual reporter assay, thereby facilitating high throughput screening.

### The influences of substrate and enzyme concentrations on the signal half-life

Signal stability directly impacts experimental accuracy and repeatability, with signal half-life serving as a key metric for evaluating such stability. Luciferase signal half-life is influenced by two main factors, substrate concentration (**Figure 1**) and luciferase concentration (**Figure 2**, **Table 1**), which has not been determined systematically.

Substrate concentration has complex and varied effects on the signal half-lives of different luciferases. First, substrate concentrations should not be excessively low, as this can reduce signal half-life due to insufficient substrate availability. Theoretically, when substrate concentration is saturated, enzyme activity should be relatively stable, with a longer signal half-life. However, none of the four luciferases tested met the expectations. The signal half-lives of both RLuc (**Figure 1A**) and GLuc (**Figure 1B**) declined as substrate concentrations increased, which may be attributed to inhibition by the reaction product coelenteramide (Hart et al., 1979; Verhaegent and Christopoulos, 2002). In contrast, both iLuc (**Figure 1C**) and NanoLuc (**Figure 1D**) exhibited nearly constant signal half-lives across the tested substrate range, despite unsaturated substrate concentration.

In contrast to the variable effects of substrate concentration on signal half-life, signal half-life decreased as luciferase concentration increased (**Table 1**). This phenomenon may arise from a disrupted balance between substrate and enzyme concentration. Therefore, it is recommend to detect luciferase activity at lower enzyme concentration through dilution.

### Standard curve is required for precise protein quantification

Linearity is the basis of protein quantification of reporters. Signal intensity is only positively correlated with protein quantity but does not equate to it. However, the standard curves for protein quantifications are often absent in most current reports, most articles used signal intensity directly as a proxy for protein quantity.

The cost-effective dual reporters, NanoLuc and GFP, exhibit linearity within our current detectable ranges, with their maximum values approaching the threshold limits of the used spectrometer, 1×10^7^ for chemiluminescent proteins and 6×10^4^ for fluorescent proteins. In contrast, most reporters display linearity only at lower protein concentrations (**Figures 2**, **4**). Therefore, the key determinant of a reporter’s linear range is the highest detected reporter concentration or signal, rather than the order of magnitude spanned by reporter concentrations through serial dilution (Sherf et al., 1996; Verhaegent and Christopoulos, 2002). Otherwise, the wrong conclusions may be drawn. For example, GLuc was initially reported as a linear reporter with a maximum RLU of ~1×10^6^ (Verhaegent and Christopoulos, 2002). However, our results indicate that it shows linearity only when its RLU is below 3×10^6^ (**Figure 2F**).

Additionally, luciferase autofluorescence values are usually about 1×10^4^~3×10^4^ in our research. If RLU value is at least one order of magnitude greater than the autofluorescence value, the luciferase protein content can be directly assessed using RLU value. If the RLU value is low, the influence of substrate autofluorescence should not be ignored. On the other hand, it is important to note that it is insufficient for the reporter activity to fall within the linear range of the standard curve. Because some reporters show reduced activities at high concentrations, such as ZsGreen (**Figure 4B**) and TagRFP-T (**Figure 4C**). For such reporters, reassessment via serial dilution is necessary. All these drawbacks can be addressed through the use of standard curves.

### The workflow of the protein quantification experimental procedures

**Figure 6:**
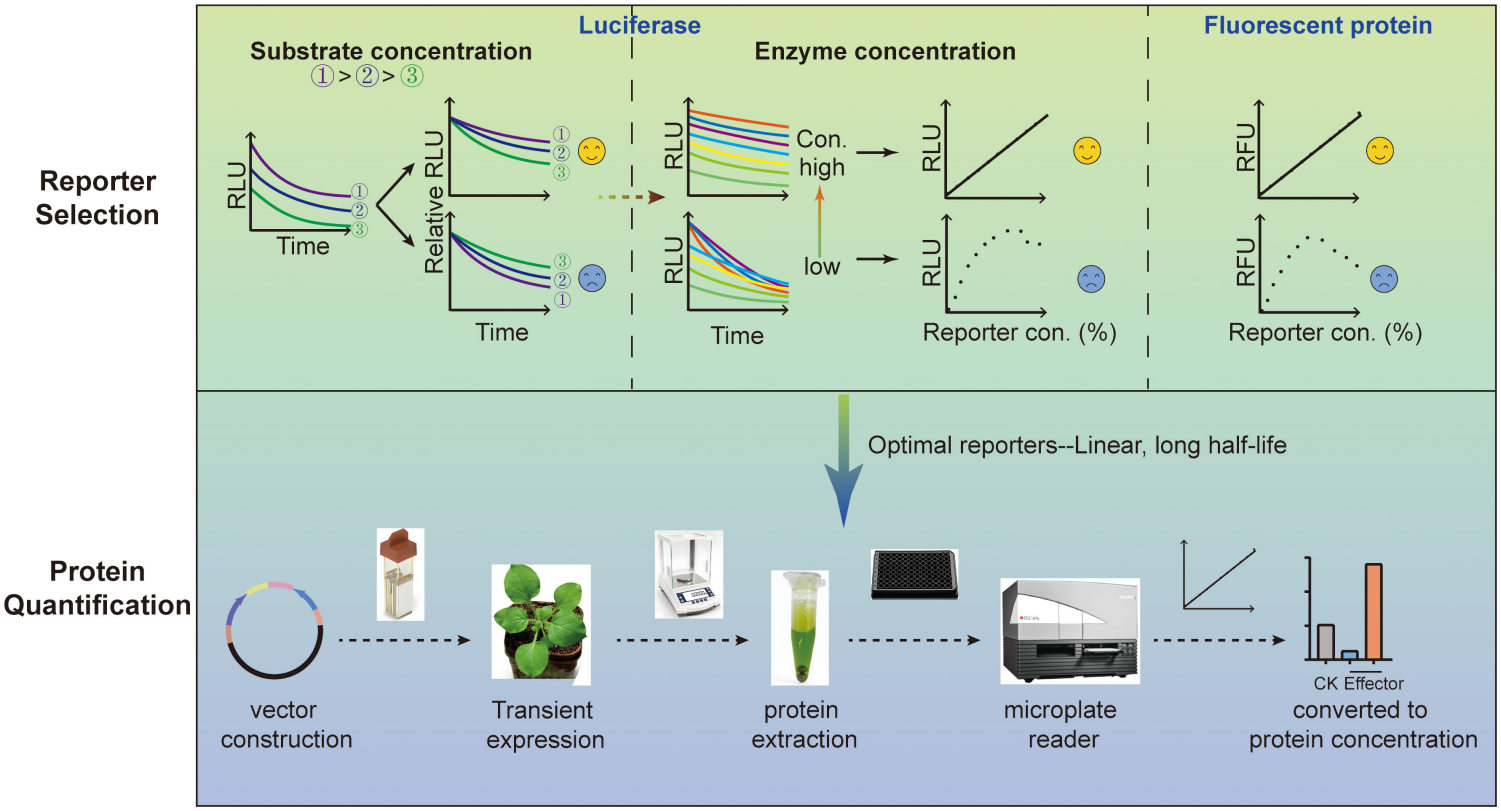
Workflow of the protein quantification experimental procedures. The first section is reporter selection, which contains at least two types of reporters. For luciferase, the optimal substrate concentration must be tested first according to the half-life. Then the standard curve should be constructed through serial dilution. Linear reporter is the basis for protein quantification. For fluorescent protein, the standard curve should be similarly constructed through serial dilution. Finally, linear and stable reporters were selected as optimal reporters for subsequent protein quantification. Subsequent to vector construction, agrobacterium transformation, and transient protein expression, plant materials with equal fresh weight are recommended for sampling to ensure similar lysis efficiency. Subsequently, total proteins were extracted, and RLU or RFU values are measured by a microplate reader. Finally, the values are converted to relative protein concentrations according to the standard curves to characterize the function of the effector.

To illustrate the procedure, a workflow diagram was constructed (**Figure 6**). Briefly, for luciferase, optimal substrate concentration should first be screened at a relative low luciferase concentration via analyzing the time course curves of RLU signals, otherwise erroneous conclusions might be concluded due to insufficient substrate availability when luciferase concentration is excessively high. The signal of desired reporter should exhibit greater stability at elevated substrate concentrations. Based on the magnitude of the RLU signals and substrate cost, RLU values at higher luciferase concentrations should also be measured with the selected or higher substrate concentrations to avoid standard curve inaccuracies caused by substrate insufficiency. For fluorescent protein, the optimal detection parameters, particularly the gain manual setting, should be determined to avoid the system error “over”, a frequently occurred phenomenon where the value exceeds the detection range.

Following the screening of the optimal substrate concentration, establishment of standard curves, and selection of optimal reporters, the dual reporter system was established. Subsequent to vector construction, electroporation-mediated Agrobacterium transformation for transient expression, plant materials with equal fresh weight were recommended for sampling to ensure consistent lysis efficiency. Total proteins were then extracted, and the RLU or RFU values were measured using a microplate reader. Finally, the obtained signal values were converted to relative protein concentration based on the standard curves to reduce the interference from background, such as autofluorescence.

## Conclusions

This study first systematically characterized commonly used or potential reporters for relative protein quantification. Through systematic testing of the signal half-lives of luciferases at different substrate concentrations, we found complex effects of substrate concentration on luciferase activity. For example, the signal half-lives of RLuc and GLuc decreased with increasing substrate concentration, attributed to inhibition by their reaction products; in contrast, those of iLuc and NanoLuc remain nearly constant. Then, we systematically evaluated the influence of enzyme concentrations. For all the tested luciferases, their half-lives exhibit a decreasing trend with their enzyme concentrations increasing. Therefore, for luciferase assays, it is advisable to select appropriate substrate concentrations and relatively low enzyme concentrations.

Based on the signal stability and operational simplicity, NanoLuc and GFP are recommended as preferred reporters for protein quantification. The NanoLuc-GFP system showed high stability, which can largely reduce the interference caused by biological variability and dramatic environmental fluctuations. Moreover, compared with the conventional FLuc/RLuc system, the GFP/NanoLuc system not only costs less than one-tenth as much, but also exhibits greater sensitivity.

## Data Availability

The original contributions presented in the study are included in the article/**Supplementary Material**. Further inquiries can be directed to the corresponding author.
