# Supplementary material for: A cost-effective dual reporter system in Nicotiana benthamiana
Source: Front Plant Sci. 2026 Feb 3;17:1732683. doi: 10.3389/fpls.2026.1732683 (PMC12909557; doi:10.3389/fpls.2026.1732683)
Supplement: Supplementary file 1 [file DataSheet1.docx]

**Materials and methods**

**Absolute quantification of linear reporter proteins**

Proteins from three biological repeats (R1, R2, R3) were seperated by SDS-PAGE and stained with a Coomassie Brilliant Blue staining buffer (0.025% coomassie blue R250, 40% methanol, 7% acetic acid) for 30 min at room temperature. Then the stained gels were destained twice with a destaining buffer (0.25 M KCl) at room temperature. The detailed protein quantification of the three tested linear reporters was as follows.

RLuc: The protein extraction solutions containing RLuc were subjected to 5-fold (R1, R2) or 10-fold (R3) dilution with the protein extraction buffer. The diluted solutions showed RLU values of ~7,000,000 (Supplementary Figure 2D), which was defined as the 100% relative protein concentration in Figure 2A. For SDS-PAGE analysis (Supplementary Figure 2A), undiluted protein extraction solutions were loaded and annotated as 5× (R1, R2) and 10× (R3) loading amounts. Additionally, two fold colume solutions were loaded, yielding 10× (R1, R2) and 20× (R3) loading amounts. Based on the standard bovine serum albumin (BSA), the estimated RLuc protein levels were approximately 75 ng for both R1 (10×) and R2 (10×), and 150 ng for R3 (20×). The molecular weight of RLuc is approximately 36 kDa. Using these parameters, the concentration (*C_R_*) of the undiluted RLuc protein solution (R1) was caculated via the following equation:

12 × 10^-6^ L × *C_R_* mol/L × 36,000 g/mol = 75 × 10^-9^ g

The derived *C_R_* value of R1 solution was 170 nM. So the 100% relative protein concentration of RLuc in Figure 2A was approximately 17 nM.

NanoLuc: The protein extraction solutions containing NanoLuc were subjected to 2000-fold (R1, R2, R3) dilution with the protein extraction buffer. The diluted solutions showed RLU values of ~8,000,000 (Supplementary Figure 2D), which was defined as the 80% relative protein concentration in Figure 2D. For SDS-PAGE analysis (Supplementary Figure 2B), undiluted protein extraction solutions were loaded and annotated as 2000× and 4000× (R3) loading amounts. Based on the standard BSA, the estimated NanoLuc protein levels were approximately 150 ng for R1 (4000×), and 200 ng for both R2 (4000×) and R3 (4000×). The molecular weight of NanoLuc is approximately 19 kDa. Using these parameters, the concentration (*C_N_*) of the undiluted NanoLuc protein solution (R2) was caculated via the following equation:

12 × 10^-6^ L × *C_N_* mol/L × 19,000 g/mol = 200 × 10^-9^ g

The derived *C_N_* value of R2 solution was 880 nM. So the 100% relative protein concentration of NanoLuc in Figure 2D was approximately 0.275 nM.

GFP: Purified His-GFP protein (4500 ng/μl) expressed in *E.coli* were employed as the standard for absolute protein quantification of GFP. The protein concentration was confirmed via the Bradford assay, as detailed in Supplementary Figure S2C. A 143 fold dilution of the His-GFP yielded RFU values of ~60,000 (Supplementary Figure 2D), which was defined as the 100% relavtive protein concentration in Figure 4A. The molecular weight of His-GFP is approximately 28 kDa. Using these parameters, the concentration (*C_G_*) corresponding to the 100% relavtive protein concentration of GFP was caculated via the following equation:

1 × 10^-3^ L × *C_G_* mol/L × 28,000 g/mol = 4500 × 7 × 10^-9^ g

The derived *C_G_* value corresponding to the 100% relavtive protein concentration of GFP in Figure 4A was 1125 nM.


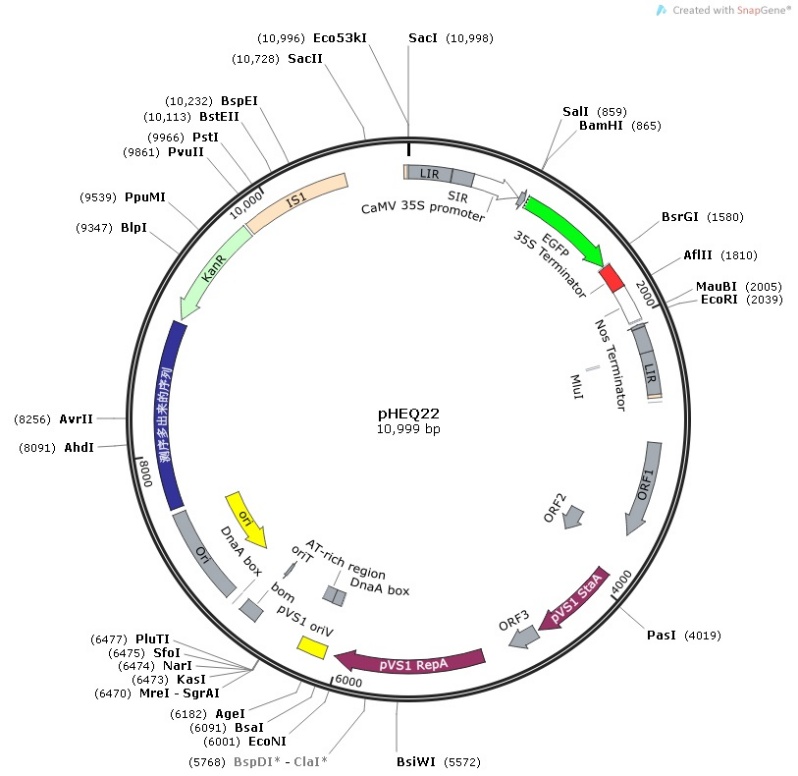


Full sequence of pHEQ22-GFP plasmid:

LOCUS Exported 10999 bp ds-DNA circular SYN 1-Jan-2026

SOURCE synthetic DNA construct

REFERENCE 1 (bases 1 to 10999)

FEATURES Location/Qualifiers

source 1..10999

/organism="synthetic DNA construct"

/mol_type="other DNA"

misc_feature 1..305

/note="LIR"

misc_feature 312..461

/note="SIR"

promoter 462..807

/note="CaMV 35S promoter"

/note="strong constitutive promoter from cauliflower mosaic

virus"

misc_feature 815..855

/note="AtPsaK 5'-UTR"

CDS 871..1590

/codon_start=1

/product="enhanced GFP"

/note="EGFP"

/note="mammalian codon-optimized"

/translation="MVSKGEELFTGVVPILVELDGDVNGHKFSVSGEGEGDATYGKLTL

KFICTTGKLPVPWPTLVTTFTYGVQCFSRYPDHMKQHDFFKSAMPEGYVQERTIFFKDD

GNYKTRAEVKFEGDTLVNRIELKGIDFKEDGNILGHKLEYNYNSHNVYIMADKQKNGIK

VNFKIRHNIEDGSVQLADHYQQNTPIGDGPVLLPDNHYLSTQSALSKDPNEKRDHMVLL

EFVTAAGITHGMDELYK"

misc_feature 1591..1596

/note="StuI"

terminator 1601..1780

/label="35S Terminator"

/note="35S Terminator"

terminator 1781..2033

/note="Nos Terminator"

polyA_signal 2093..2269

/note="CaMV poly(A) signal"

/note="cauliflower mosaic virus polyadenylation signal"

misc_feature 2270..2574

/note="LIR"

misc_feature 2575..2599

/note="RB T-DNA repeat"

/note="right border repeat from nopaline C58 T-DNA"

misc_feature 2915..3601

/note="ORF1"

/note="not required for stable maintenance"

misc_feature 3598..3813

/note="ORF2"

/note="not required for stable maintenance"

CDS 3900..4529

/codon_start=1

/product="stability protein from the Pseudomonas plasmid

pVS1 (Heeb et al., 2000)

partioning protein"

/note="pVS1 StaA"

/translation="MKVIAVLNQKGGSGKTTIATHLARALQLAGADVLLVDSDPQGSAR

DWAAVREDQPLTVVGIDRPTIDRDVKAIGRRDFVVIDGAPQAADLAVSAIKAADFVLIP

VQPSPYDIWATADLVELVKQRIEVTDGRLQAAFVVSRAIKGTRIGGEVAEALAGYELPI

LESRITQRVSYPGTAAAGTTVLESEPEGDAAREVQALAAEIKSKLI"

misc_feature 4550..4765

/note="ORF3"

/note="not required for stable maintenance"

CDS 4963..6030

/codon_start=1

/product="replication protein from the Pseudomonas plasmid

pVS1 (Heeb et al., 2000)"

/note="pVS1 RepA"

/translation="GRKPSGPVQIGAALGDDLVEKLKAAQAAQRQRIEAEARPGESWQA

AADRIRKESRQPPAAGAPSIRKPPKGDEQPDFFVPMLYDVGTRDSRSIMDVAVFRLSKR

DRRAGEVIRYELPDGHVEVSAGPAGMASVWDYDLVLMAVSHLTESMNRYREGKGDKPGR

VFRPHVADVLKFCRRADGGKQKDDLVETCIRLNTTHVAMQRTKKAKNGRLVTVSEGEAL

ISRYKIVKSETGRPEYIEIELADWMYREITEGKNPDVLTVHPDYFLIDPGIGRFLYRLA

RRAAGKAEARWLFKTIYERSGSAGEFKKFCFTVRKLIGSNDLPEYDLKEEAGQAGPILV

MRYRNLIEGEASAGS"

rep_origin 6096..6290

/note="pVS1 oriV"

/note="origin of replication for the Pseudomonas plasmid

pVS1 (Heeb et al., 2000)"

misc_feature 6096..6103

/note="DnaA box"

misc_feature 6107..6190

/note="21bp repeats"

misc_feature 6191..6282

/note="AT-rich region"

misc_feature 6283..6290

/note="DnaA box"

misc_feature 6634..6774

/note="bom"

/note="basis of mobility region from pBR322"

misc_feature complement(6655..6682)

/note="oriT"

misc_feature 6832..6839

/note="DnaA box"

misc_feature 6897..7571

/note="Ori"

rep_origin complement(6960..7548)

/direction=LEFT

/note="ori"

/note="high-copy-number ColE1/pMB1/pBR322/pUC origin of

replication"

CDS complement(8973..9767)

/codon_start=1

/gene="aphA-3"

/product="aminoglycoside phosphotransferase"

/note="KanR"

/note="confers resistance to kanamycin"

/translation="MAKMRISPELKKLIEKYRCVKDTEGMSPAKVYKLVGENENLYLKM

TDSRYKGTTYDVEREKDMMLWLEGKLPVPKVLHFERHDGWSNLLMSEADGVLCSEEYED

EQSPEKIIELYAECIRLFHSIDISDCPYTNSLDSRLAELDYLLNNDLADVDCENWEEDT

PFKDPRELYDFLKTEKPEEELVFSHGDLGDSNIFVKDGKVSGFIDLGRSGRADKWYDIA

FCVRSIREDIGEEQYVELFFDLLGIKPDWEKIKYYILLDELF"

mobile_element 9787..10554

/mobile_element_type="insertion sequence:IS1"

/note="IS1"

/note="prokaryotic transposable element"

misc_feature 10969..10993

/note="LB T-DNA repeat"

/note="left border repeat from nopaline C58 T-DNA"

misc_feature 10994..10999

/note="SacI"

ORIGIN

1 gtggttgtga ggcgcgccac gcgtctccga ggggttgcct caaactctat cttataaccg

61 gcgtggaggc atggaggcaa gggcattttg gtaatttaag tagttagtgg aaaatgacgt

121 catttactta aagacgaagt cttgcgacaa ggggggccca cgccgaattt taatattacc

181 ggcgtggccc caccttatcg cgagtgcttt agcacgagcg gtccagattt aaagtagaaa

241 agttcccgcc cactagggtt aaaggtgttc acactataaa agcatatacg atgtgatggt

301 atttgcccgg gaatgattat tttatgaata tatttcattg tgcaagtaga tagaaattac

361 atatgttaca taacacacga aataaacaaa aaaacacaat ccaaaacaaa caccccaaac

421 aaaataacac tatatatatc ctcgtatgag gagaggcacg ttgagacttt tcaacaaagg

481 gtaatttcgg gaaacctcct cggattccat tgcccagcta tctgtcactt catcgaaagg

541 acagtagaaa aggaaggtgg ctcctacaaa tgccatcatt gcgataaagg aaaggctatc

601 attcaagatg cctctgccga cagtggtccc aaagatggac ccccacccac gaggagcatc

661 gtggaaaaag aagacgttcc aaccacgtct tcaaagcaag tggattgatg tgacatctcc

721 actgacgtaa gggatgacgc acaatcccac tatccttcgc aagacccttc ctctatataa

781 ggaagttcat ttcatttgga gaggacagcc caagtcgcac aagaaaataa aagatttgta

841 gaatcaacta agaaacttgt cgacggatcc atggtgagca agggcgagga gctgttcacc

901 ggggtggtgc ccatcctggt cgagctggac ggcgacgtaa acggccacaa gttcagcgtg

961 tccggcgagg gcgagggcga tgccacctac ggcaagctga ccctgaagtt catctgcacc

1021 accggcaagc tgcccgtgcc ctggcccacc ctcgtgacca ccttcaccta cggcgtgcag

1081 tgcttcagcc gctaccccga ccacatgaag cagcacgact tcttcaagtc cgccatgccc

1141 gaaggctacg tccaggagcg caccatcttc ttcaaggacg acggcaacta caagacccgc

1201 gccgaggtga agttcgaggg cgacaccctg gtgaaccgca tcgagctgaa gggcatcgac

1261 ttcaaggagg acggcaacat cctggggcac aagctggagt acaactacaa cagccacaac

1321 gtctatatca tggccgacaa gcagaagaac ggcatcaagg tgaacttcaa gatccgccac

1381 aacatcgagg acggcagcgt gcagctcgcc gaccactacc agcagaacac ccccatcggc

1441 gacggccccg tgctgctgcc cgacaaccac tacctgagca cccagtccgc cctgagcaaa

1501 gaccccaacg agaagcgcga tcacatggtc ctgctggagt tcgtgaccgc cgccgggatc

1561 actcacggca tggacgagct gtacaagtaa aggccttcga tctccataat aatgtgtgag

1621 tagttcccag ataagggaat tagggttcct atagggtttc gctcatgtgt tgagcatata

1681 agaaaccctt agtatgtatt tgtatttgta aaatacttct atcaataaaa tttctaattc

1741 ctaaaaccaa aatccagtac taaaatccag atccccgtcg gatcgttcaa acatttggca

1801 ataaagtttc ttaagattga atcctgttgc cggtcttgcg atgattatca tataatttct

1861 gttgaattac gttaagcatg taataattaa catgtaatgc atgacgttat ttatgagatg

1921 ggtttttatg attagagtcc cgcaattata catttaatac gcgatagaaa acaaaatata

1981 gcgcgcaaac taggataaat tatcgcgcgc ggtgtcatct atgttactag atccccggga

2041 attcggtacg ctgaaatcac cagtctctct ctacaaatct atctctctct attttctcca

2101 taaataatgt gtgagtagtt tcccgataag ggaaattagg gttcttatag ggtttcgctc

2161 atgtgttgag catataagaa acccttagta tgtatttgta tttgtaaaat acttctatca

2221 ataaaatttc taattcctaa aaccaaaatc cagtactaaa atccagatcg tggttgtgag

2281 gcgcgccacg cgtctccgag gggttgcctc aaactctatc ttataaccgg cgtggaggca

2341 tggaggcaag ggcattttgg taatttaagt agttagtgga aaatgacgtc atttacttaa

2401 agacgaagtc ttgcgacaag gggggcccac gccgaatttt aatattaccg gcgtggcccc

2461 accttatcgc gagtgcttta gcacgagcgg tccagattta aagtagaaaa gttcccgccc

2521 actagggtta aaggtgttca cactataaaa gcatatacga tgtgatggta tttgtgacag

2581 gatatattgg cgggtaaacc taagagaaaa gagcgtttat tagaataatc ggatatttaa

2641 aagggcgtga aaaggtttat ccgttcgtcc atttgtatgt gcatgccaac cacagggttc

2701 ccctcgggat caaagtactt tgatccaacc cctccgctgc tatagtgcag tcggcttctg

2761 acgttcagtg cagccgtctt ctgaaaacga catgtcgcac aagtcctaag ttacgcgaca

2821 ggctgccgcc ctgccctttt cctggcgttt tcttgtcgcg tgttttagtc gcataaagta

2881 gaatacttgc gactagaacc ggagacatta cgccatgaac aagagcgccg ccgctggcct

2941 gctgggctat gcccgcgtca gcaccgacga ccaggacttg accaaccaac gggccgaact

3001 gcacgcggcc ggctgcacca agctgttttc cgagaagatc accggcacca ggcgcgaccg

3061 cccggagctg gccaggatgc ttgaccacct acgccctggc gacgttgtga cagtgaccag

3121 gctagaccgc ctggcccgca gcacccgcga cctactggac attgccgagc gcatccagga

3181 ggccggcgcg ggcctgcgta gcctggcaga gccgtgggcc gacaccacca cgccggccgg

3241 ccgcatggtg ttgaccgtgt tcgccggcat tgccgagttc gagcgttccc taatcatcga

3301 ccgcacccgg agcgggcgcg aggccgccaa ggcccgaggc gtgaagtttg gcccccgccc

3361 taccctcacc ccggcacaga tcgcgcacgc ccgcgagctg atcgaccagg aaggccgcac

3421 cgtgaaagag gcggctgcac tgcttggcgt gcatcgctcg accctgtacc gcgcacttga

3481 gcgcagcgag gaagtgacgc ccaccgaggc caggcggcgc ggtgccttcc gtgaggacgc

3541 attgaccgag gccgacgccc tggcggccgc cgagaatgaa cgccaagagg aacaagcatg

3601 aaaccgcacc aggacggcca ggacgaaccg tttttcatta ccgaagagat cgaggcggag

3661 atgatcgcgg ccgggtacgt gttcgagccg cccgcgcacg tctcaaccgt gcggctgcat

3721 gaaatcctgg ccggtttgtc tgatgccaag ctggcggcct ggccggccag cttggccgct

3781 gaagaaaccg agcgccgccg tctaaaaagg tgatgtgtat ttgagtaaaa cagcttgcgt

3841 catgcggtcg ctgcgtatat gatgcgatga gtaaataaac aaatacgcaa ggggaacgca

3901 tgaaggttat cgctgtactt aaccagaaag gcgggtcagg caagacgacc atcgcaaccc

3961 atctagcccg cgccctgcaa ctcgccgggg ccgatgttct gttagtcgat tccgatcccc

4021 agggcagtgc ccgcgattgg gcggccgtgc gggaagatca accgctaacc gttgtcggca

4081 tcgaccgccc gacgattgac cgcgacgtga aggccatcgg ccggcgcgac ttcgtagtga

4141 tcgacggagc gccccaggcg gcggacttgg ctgtgtccgc gatcaaggca gccgacttcg

4201 tgctgattcc ggtgcagcca agcccttacg acatatgggc caccgccgac ctggtggagc

4261 tggttaagca gcgcattgag gtcacggatg gaaggctaca agcggccttt gtcgtgtcgc

4321 gggcgatcaa aggcacgcgc atcggcggtg aggttgccga ggcgctggcc gggtacgagc

4381 tgcccattct tgagtcccgt atcacgcagc gcgtgagcta cccaggcact gccgccgccg

4441 gcacaaccgt tcttgaatca gaacccgagg gcgacgctgc ccgcgaggtc caggcgctgg

4501 ccgctgaaat taaatcaaaa ctcatttgag ttaatgaggt aaagagaaaa tgagcaaaag

4561 cacaaacacg ctaagtgccg gccgtccgag cgcacgcagc agcaaggctg caacgttggc

4621 cagcctggca gacacgccag ccatgaagcg ggtcaacttt cagttgccgg cggaggatca

4681 caccaagctg aagatgtacg cggtacgcca aggcaagacc attaccgagc tgctatctga

4741 atacatcgcg cagctaccag agtaaatgag caaatgaata aatgagtaga tgaattttag

4801 cggctaaagg aggcggcatg gaaaatcaag aacaaccagg caccgacgcc gtggaatgcc

4861 ccatgtgtgg aggaacgggc ggttggccag gcgtaagcgg ctgggttgtc tgccggccct

4921 gcaatggcac tggaaccccc aagcccgagg aatcggcgtg acggtcgcaa accatccggc

4981 ccggtacaaa tcggcgcggc gctgggtgat gacctggtgg agaagttgaa ggccgcgcag

5041 gccgcccagc ggcaacgcat cgaggcagaa gcacgccccg gtgaatcgtg gcaagcggcc

5101 gctgatcgaa tccgcaaaga atcccggcaa ccgccggcag ccggtgcgcc gtcgattagg

5161 aagccgccca agggcgacga gcaaccagat tttttcgttc cgatgctcta tgacgtgggc

5221 acccgcgata gtcgcagcat catggacgtg gccgttttcc gtctgtcgaa gcgtgaccga

5281 cgagctggcg aggtgatccg ctacgagctt ccagacgggc acgtagaggt ttccgcaggg

5341 ccggccggca tggccagtgt gtgggattac gacctggtac tgatggcggt ttcccatcta

5401 accgaatcca tgaaccgata ccgggaaggg aagggagaca agcccggccg cgtgttccgt

5461 ccacacgttg cggacgtact caagttctgc cggcgagccg atggcggaaa gcagaaagac

5521 gacctggtag aaacctgcat tcggttaaac accacgcacg ttgccatgca gcgtacgaag

5581 aaggccaaga acggccgcct ggtgacggta tccgagggtg aagccttgat tagccgctac

5641 aagatcgtaa agagcgaaac cgggcggccg gagtacatcg agatcgagct agctgattgg

5701 atgtaccgcg agatcacaga aggcaagaac ccggacgtgc tgacggttca ccccgattac

5761 tttttgatcg atcccggcat cggccgtttt ctctaccgcc tggcacgccg cgccgcaggc

5821 aaggcagaag ccagatggtt gttcaagacg atctacgaac gcagtggcag cgccggagag

5881 ttcaagaagt tctgtttcac cgtgcgcaag ctgatcgggt caaatgacct gccggagtac

5941 gatttgaagg aggaggcggg gcaggctggc ccgatcctag tcatgcgcta ccgcaacctg

6001 atcgagggcg aagcatccgc cggttcctaa tgtacggagc agatgctagg gcaaattgcc

6061 ctagcagggg aaaaaggtcg aaaaggtctc tttcctgtgg atagcacgta cattgggaac

6121 ccaaagccgt acattgggaa ccggaacccg tacattggga acccaaagcc gtacattggg

6181 aaccggtcac acatgtaagt gactgatata aaagagaaaa aaggcgattt ttccgcctaa

6241 aactctttaa aacttattaa aactcttaaa acccgcctgg cctgtgcata actgtctggc

6301 cagcgcacag ccgaagagct gcaaaaagcg cctacccttc ggtcgctgcg ctccctacgc

6361 cccgccgctt cgcgtcggcc tatcgcggcc gctggccgct caaaaatggc tggcctacgg

6421 ccaggcaatc taccagggcg cggacaagcc gcgccgtcgc cactcgaccg ccggcgccca

6481 catcaaggca ccctgcctcg cgcgtttcgg tgatgacggt gaaaacctct gacacatgca

6541 gctcccggag acggtcacag cttgtctgta agcggatgcc gggagcagac aagcccgtca

6601 gggcgcgtca gcgggtgttg gcgggtgtcg gggcgcagcc atgacccagt cacgtagcga

6661 tagcggagtg tatactggct taactatgcg gcatcagagc agattgtact gagagtgcac

6721 catatgcggt gtgaaatacc gcacagatgc gtaaggagaa aataccgcat caggcgctct

6781 tccgcttcct cgctcactga ctcgctgcgc tcggtcgttc ggctgcggcg agcggtatca

6841 gctcactcaa aggcggtaat acggttatcc acagaatcag gggataacgc aggaaagaac

6901 atgtgagcaa aaggccagca aaaggccagg aaccgtaaaa aggccgcgtt gctggcgttt

6961 ttccataggc tccgcccccc tgacgagcat cacaaaaatc gacgctcaag tcagaggtgg

7021 cgaaacccga caggactata aagataccag gcgtttcccc ctggaagctc cctcgtgcgc

7081 tctcctgttc cgaccctgcc gcttaccgga tacctgtccg cctttctccc ttcgggaagc

7141 gtggcgcttt ctcatagctc acgctgtagg tatctcagtt cggtgtaggt cgttcgctcc

7201 aagctgggct gtgtgcacga accccccgtt cagcccgacc gctgcgcctt atccggtaac

7261 tatcgtcttg agtccaaccc ggtaagacac gacttatcgc cactggcagc agccactggt

7321 aacaggatta gcagagcgag gtatgtaggc ggtgctacag agttcttgaa gtggtggcct

7381 aactacggct acactagaag gacagtattt ggtatctgcg ctctgctgaa gccagttacc

7441 ttcggaaaaa gagttggtag ctcttgatcc ggcaaacaaa ccaccgctgg tagcggtggt

7501 ttttttgttt gcaagcagca gattacgcgc agaaaaaaag gatctcaaga agatcctttg

7561 atcttttcta cggggtctga cgctcagtgg aacgaaaact cacgttaagg gctgagagat

7621 cccctcataa tttccccaaa gcgtaaccat gtgtgaataa attttgagct agtagggttg

7681 cagccacgag taagtcttcc cttgttattg tgtagccaga atgccgcaaa acttccatgc

7741 ctaagcgaac tgttgagagt acgtttcgat ttctgactgt gttagcctgg aagtgcttgt

7801 cccaaccttg tttctgagca tgaacgcccg caagccaaca tgttagttga agcatcaggg

7861 cgattagcag catgatatca aaacgctctg agctgctcgt tcggctatgg cgtaggccta

7921 gtccgtaggc aggacttttc aagtctcgga aggtttcttc aatctgcatt cgcttcgaat

7981 agatattaac aagttgtttg ggtgttcgaa tttcaacagg taagttagtt gctagaatcc

8041 atggctcctt tgccgacgct gagtagattt taggtgacgg gtggtgacaa tgagtccgtg

8101 tcgagcgctg attttttcgg cctttagagc gagatttata caatagaatt tggcatgaga

8161 ttggattgct tttagtcagc ctcttatagc ctaaagtctt tgagtgacta gatgacatat

8221 catgtaagtt gctgataggt ttccagtttt ccgctcctag gtctgcatat tgtacttttc

8281 ctcttactcg acttaaccag taccaaccca gcttctcaac ggatttatac catggcactt

8341 taaagccagc atcactgaca atgagcggtg tggtgttact cggtagaatg ctcgcaaggt

8401 cggctagaaa ttggtcatga gctttctttg aacattgctc tgaaagcggg aacgctttct

8461 cataaagagt aacagaacga ccgtgtagtg cgactgaagc tcgcaatacc ataagccgtt

8521 tttgctcacg gatatcagac cagtcaacaa gtacaatggg catcgtattg cccgaacaga

8581 taaagctagc atgccaacgg tatacagcga gtcgctcttt gtggaggtga cgattaccta

8641 acaatcggtc gattcgtttg atgttatgtt ttgttctcgc tttggttggc aggttacggc

8701 caagttcggt aagagtgaga gttttacagt caagtaaggc gtggcaagcc aacgttaagc

8761 tgttgagtcg ttttaagtgt aattcggggc agaattggta aagagagtcg tgtaaaatat

8821 cgagttcgca cattttgttg tctgattatt gatttttggc gaaaccattt gatcatatga

8881 caagatgtgt atctacctta acttaatgat tttgataaaa atcattaggg gattcatcag

8941 cgttaaggga ttttggtcat gcattctagg tactaaaaca attcatccag taaaatataa

9001 tattttattt tctcccaatc aggcttgatc cccagtaagt caaaaaatag ctcgacatac

9061 tgttcttccc cgatatcctc cctgatcgac cggacgcaga aggcaatgtc ataccacttg

9121 tccgccctgc cgcttctccc aagatcaata aagccactta ctttgccatc tttcacaaag

9181 atgttgctgt ctcccaggtc gccgtgggaa aagacaagtt cctcttcggg cttttccgtc

9241 tttaaaaaat catacagctc gcgcggatct ttaaatggag tgtcttcttc ccagttttcg

9301 caatccacat cggccagatc gttattcagt aagtaatcca attcggctaa gcggctgtct

9361 aagctattcg tatagggaca atccgatatg tcgatggagt gaaagagcct gatgcactcc

9421 gcatacagct cgataatctt ttcagggctt tgttcatctt catactcttc cgagcaaagg

9481 acgccatcgg cctcactcat gagcagattg ctccagccat catgccgttc aaagtgcagg

9541 acctttggaa caggcagctt tccttccagc catagcatca tgtccttttc ccgttccaca

9601 tcataggtgg tccctttata ccggctgtcc gtcattttta aatataggtt ttcattttct

9661 cccaccagct tatatacctt agcaggagac attccttccg tatcttttac gcagcggtat

9721 ttttcgatca gttttttcaa ttccggtgat attctcattt tagccattta ttatttcctt

9781 cctcttggtg atgctgccaa cttactgatt tagtgtatga tggtgttttt gaggtgctcc

9841 agtggcttct gtttctatca gctgtccctc ctgttcagct actgacgggg tggtgcgtaa

9901 cggcaaaagc accgccggac atcagcgcta tctctgctct cactgccgta aaacatggca

9961 actgcagttc acttacaccg cttctcaacc cggtacgcac cagaaaatca ttgatatggc

10021 catgaatggc gttggatgcc gggcaacagc ccgcattatg ggcgttggcc tcaacacgat

10081 tttacgtcac ttaaaaaact caggccgcag tcggtaacct cgcgcataca gccgggcagt

10141 gacgtcatcg tctgcgcgga aatggacgaa cagtggggct atgtcggggc taaatcgcgc

10201 cagcgctggc tgttttacgc gtatgacagt ctccggaaga cggttgttgc gcacgtattc

10261 ggtgaacgca ctatggcgac gctggggcgt cttatgagcc tgctgtcacc ctttgacgtg

10321 gtgatatgga tgacggatgg ctggccgctg tatgaatccc gcctgaaggg aaagctgcac

10381 gtaatcagca agcgatatac gcagcgaatt gagcggcata acctgaatct gaggcagcac

10441 ctggcacggc tgggacggaa gtcgctgtcg ttctcaaaat cggtggagct gcatgacaaa

10501 gtcatcgggc attatctgaa cataaaacac tatcaataag ttggagtcat tacccttcct

10561 cttttctaca gtatttaaag ataccccaag aagctaatta taacaagacg aactccaatt

10621 cactgttcct tgcattctaa aaccttaaat accagaaaac agctttttca aagttgtttt

10681 caaagttggc gtataacata gtatcgacgg agccgatttt gaaaccgcgg tgatcacagg

10741 cagcaacgct ctgtcatcgt tacaatcaac atgctaccct ccgcgagatc atccgtgttt

10801 caaacccggc agcttagttg ccgttcttcc gaatagcatc ggtaacatga gcaaagtctg

10861 ccgccttaca acggctctcc cgctgacgcc gtcccggact gatgggctgc ctgtatcgag

10921 tggtgatttt gtgccgagct gccggtcggg gagctgttgg ctggctggtg gcaggatata

10981 ttgtggtgta aacgagctc

**Supplementary Figure 1. Map and full length of pHEQ22-GFP plasmid**


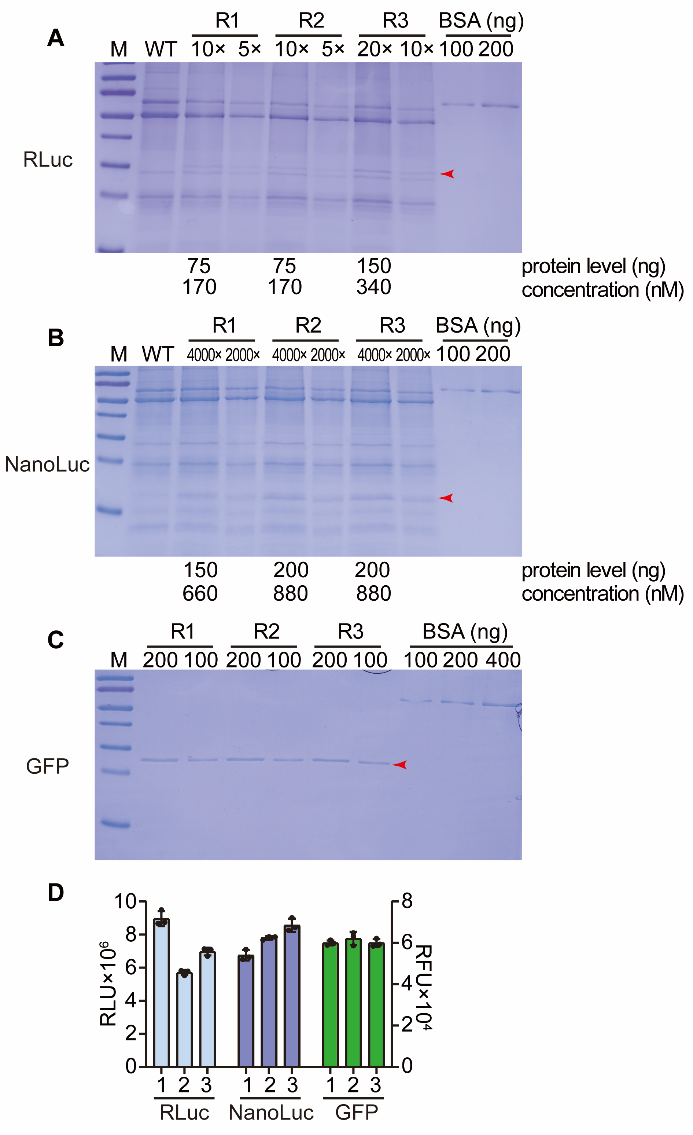


**Supplementary Figure 2. Absolute quantification of linear reporter proteins**

(A) Absolute protein quantification of RLuc. Three biological repeats (R1, R2 and R3) were included. Bovine serum albumin (BSA) served as the quantitative reference standard. The designation 5× denotes 5-fold sample dilution for RLU values detection in Supplementary Figure 2D. 10× indicates a loading volume twice that of the 5× sample. The numbers below the panel represent the predicted protein levels and corresponding concentrations. The same below in B. (B) Absolute protein quantification of NanoLuc. The designation 2000× denotes 2000-fold sample dilution for RLU values detection in Supplementary Figure 2D. 4000× indicates a loading volume twice that of the 2000× sample. (C) Verification of protein concentration for the His-GFP standard. Three technical repeates (R1, R2 and R3) were included. GFP samples with loading amounts of 200 ng and 100 ng were subjected to analysis. (D) RLU values (left y axis) and RFP values (right y axis) of diluted luciferases, RLuc and NanoLuc, and fluorescence GFP.


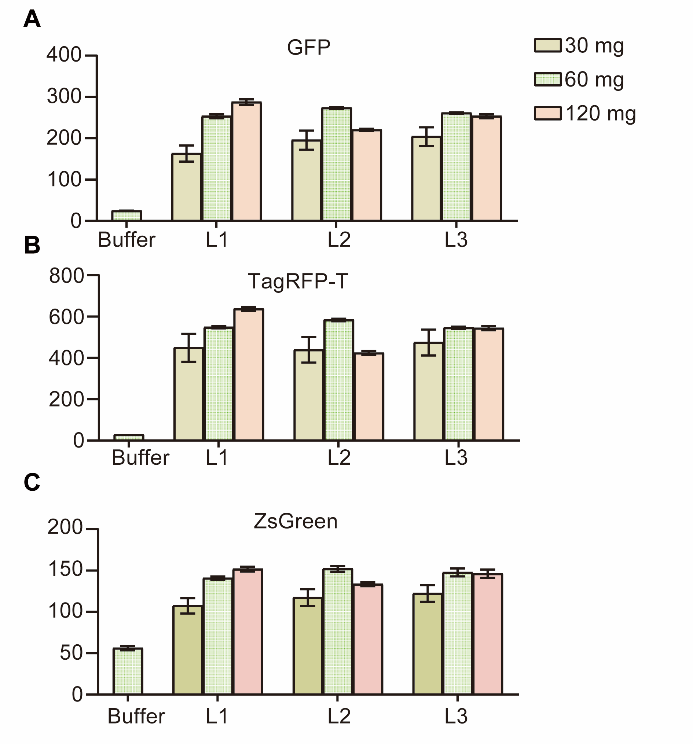


**Supplementary Figure 3. The influence of plant material amount on fluorescence background**

(A-C) Fluorescence background detection of reporter proteins, GFP (A), TagRFP-T (B) and ZsGreen (C). Leaf samples with different fresh weights were harvested from individual tobacco plants (designated L1, L2 and L3). For panels (A-C), identical protein extraction solutions were used across the experimental groups. The “Buffer” control represents protein extraction buffer without plant material.
